# Complete plastome sequencing of both living species of Circaeasteraceae (Ranunculales) reveals unusual rearrangements and the loss of the *ndh* gene family

**DOI:** 10.1186/s12864-017-3956-3

**Published:** 2017-08-09

**Authors:** Yanxia Sun, Michael J. Moore, Nan Lin, Kole F. Adelalu, Aiping Meng, Shuguang Jian, Linsen Yang, Jianqiang Li, Hengchang Wang

**Affiliations:** 1 Key Laboratory of Plant Germplasm Enhancement and Specialty Agriculture, Wuhan Botanical Garden, Chinese Academy of Sciences, Wuhan, Hubei China; 2 Department of Biology, Oberlin College, Oberlin, OH USA; 3 University of Chinese Academy of Sciences, Beijing, China; 4 South China Botanical Garden, Chinese Academy of Sciences, Guangzhou, China; 5 Hubei Key Laboratory of Shennongjia Golden Monkey Conservation Biology, Administration of Shennongjia National Park, Shennongjia, Hubei China

**Keywords:** Early-diverging eudicots, Circaeasteraceae, Plastome, Rearrangements, Gene loss, Phylogenetic analyses

## Abstract

**Background:**

Among the 13 families of early-diverging eudicots, only Circaeasteraceae (Ranunculales), which consists of the two monotypic genera *Circaeaster* and *Kingdonia,* lacks a published complete plastome sequence. In addition, the phylogenetic position of Circaeasteraceae as sister to Lardizabalaceae has only been weakly or moderately supported in previous studies using smaller data sets. Moreover, previous plastome studies have documented a number of novel structural rearrangements among early-divergent eudicots. Hence it is important to sequence plastomes from Circaeasteraceae to better understand plastome evolution in early-diverging eudicots and to further investigate the phylogenetic position of Circaeasteraceae.

**Results:**

Using an Illumina HiSeq 2000, complete plastomes were sequenced from both living members of Circaeasteraceae: *Circaeaster agrestis* and *Kingdonia uniflora*. Plastome structure and gene content were compared between these two plastomes, and with those of other early-diverging eudicot plastomes. Phylogenetic analysis of a 79-gene, 99-taxon data set including exemplars of all families of early-diverging eudicots was conducted to resolve the phylogenetic position of Circaeasteraceae.

Both plastomes possess the typical quadripartite structure of land plant plastomes. However, a large ~49 kb inversion and a small ~3.5 kb inversion were found in the large single-copy regions of both plastomes, while *Circaeaster* possesses a number of other rearrangements, particularly in the Inverted Repeat*.* In addition, *infA* was found to be a pseudogene and *accD* was found to be absent within *Circaeaster*, whereas all *ndh* genes, except for *ndhE* and *ndhJ*, were found to be either pseudogenized (*ΨndhA*, *ΨndhB*, *ΨndhD*, *ΨndhH* and *ΨndhK*) or absent (*ndhC*, *ndhF*, *ndhI* and *ndhG*) in *Kingdonia*. Circaeasteraceae was strongly supported as sister to Lardizabalaceae in phylogenetic analyses.

**Conclusion:**

The first plastome sequencing of Circaeasteraceae resulted in the discovery of several unusual rearrangements and the loss of *ndh* genes, and confirms the sister relationship between Circaeasteraceae and Lardizabalaceae. This research provides new insight to characterize plastome structural evolution in early-diverging eudicots and to better understand relationships within Ranunculales.

**Electronic supplementary material:**

The online version of this article (doi:10.1186/s12864-017-3956-3) contains supplementary material, which is available to authorized users.

## Background

The early-diverging eudicot family Circaeasteraceae (Ranunculales) sensu APG IV [1] contains only the two monotypic genera *Circaeaster* Maxim. and *Kingdonia* Balf.f. & W.W. Smith, which were historically treated as separate families (Circaeasteraceae and Kingdoniaceae) (e.g. [2–6]). *Kingdonia* has also been placed in Ranunculaceae in the past (e.g. [7–10]). *Circaeaster agrestis* Maxim. can be found in China and the Himalayas, whereas *Kingdonia uniflora* Balf.f. & W.W. Smith is endemic to China. Fossil fruits somewhat similar to those of *Circaeaster* have been reported from the mid-Albian of Virginia, USA [11, 12], while no fossil record is known for *Kingdonia*. Both species are herbs growing at high elevations, and possess the same distinctive dichotomous venation, which is very rare among angiosperms.

The Ranunculales sensu APG IV [1] form a well-supported clade comprised of seven families: Berberidaceae, Circaeasteraceae, Eupteleaceae, Lardizabalaceae, Menispermaceae, Papaveraceae and Ranunculaceae. To date, complete plastomes have been sequenced for representatives from all of these families except Circaeasteraceae [13–24]. These and plastomes from other eudicot families have helped to successfully resolve phylogenetic relationships among early-diverging eudicots, including among most families of Ranunculales (e.g.[17–19, 23, 24]). This is highly promising given that the relationships among many of these families had been poorly to moderately resolved in previous studies utilizing only a few genes (e.g. [25–38]). In previous phylogenetic studies of Ranunculales based on only a few genes, Circaeasteraceae has been resolved as sister to Lardizabalaceae, but only with weak or moderate support [25, 26, 29, 32, 36, 38, 39].

Over the past decade, knowledge of the organization and evolution of angiosperm plastomes has rapidly expanded [40, 41]. Plastomes of most flowering plants possess a typical quadripartite structure with two Inverted Repeat regions (IR_A_ and IR_B_) separating the Small and Large Single-Copy regions (SSC and LSC) [42]. Nevertheless, deviations from this canonical structure have been found with increasing frequency as the pace of plastome sequencing has exploded in recent years. For example, the length of the IR region has been found to vary significantly in some plant groups (e.g. [43–45]), and Sun et al. [23] documented six major “IR types” among 18 early-diverging eudicot plastomes, representing 12 of the 13 early-diverging eudicot families. Reconstruction of the ancestral IR gene content suggests that 18 genes were likely present in the IR region of the ancestor of eudicots [23], although representatives from Circaeasteraceae were absent from this study. Likewise, large inversions have been detected throughout the plastome in an increasing number of taxa (e.g. [46–50]). However, no obvious large inversions or rearrangements have been detected from early-diverging eudicot plastomes. Finally, gene loss (including pseudogenization) has also been found to be widespread among angiosperm plastomes, especially in species whose plastomes are highly rearranged [51].

To characterize plastome structural evolution in early-diverging eudicots and to better understand relationships within Ranunculales, we sequenced the complete plastomes of both extant species of Circaeasteraceae and included these two plastomes in a larger phylogenetic analysis including representatives of all major lineages of angiosperms. Consistent with previous work, we find that these complete plastome sequences improve support for phylogenetic relationships among Ranunculales, including the position of Circaeasteraceae. Moreover, we report several significant plastome structural changes, including a large inversion and several gene loss events.

## Results

### Plastome assemblies

Illumina paired-end sequencing produced 474,002 and 1,092,236 raw reads for *Circaeaster* and *Kingdonia*, respectively. The mean coverage of the plastome was 392.3× for *Circaeaster*, and 926.4× for *Kingdonia.* For both *Circaeaster* and *Kingdonia*, assembly yielded a single scaffold comprising the entire plastome sequence. Junction regions between the IR and Single-Copy regions were confirmed by PCR and Sanger sequencing (C8-C11 and K5-K8 in Additional file 1). Assembly statistics are presented in Table 1.

**Table 1:** Comparison of the plastid genomes of *Circaeaster agrestis* and *Kingdonia uniflora*

	*Circaeaster agrestis*	*Kingdonia uniflora*
Total plastome length (bp)	151,033	147,378
IR length (bp)	28,023	30,916
SSC length (bp)	16,857	4857
LSC length (bp)	78,130	80,689
Absent genes	*accD*	*ndhC*, *ndhF*, *ndhI*, *ndhG*
Pseudogenes	*ΨinfA*	*ΨndhA, ΨndhB, ΨndhD, ΨndhH, ΨndhK*
Overall G/C content (%)	38.2	37.8
Average depth of coverage	392.3×	926.4×
Number of plastid reads	474,002	1,092,236
Read length (bp)	125	125
Genes with one intron	*trnK-UUU*, *trnG-UCC*, *trnL-UAA*, *trnV-UAC*, *trnI-GAU*, *trnA-UGC*, *petB*, *petD*, *atpF*, *ndhA*, *ndhB*, *rpl16*, *rpoC1*, *rps16*	*trnK-UUU*, *trnG-UCC*, *trnL-UAA*, *trnV-UAC*, *trnI-GAU*, *trnA-UGC*, *petB*, *petD*, *atpF*, *rpl16*, *rpoC1*, *rps16*
Genes with two introns	*rps12*, *clpP*, *ycf3*	*rps12*, *clpP*, *ycf3*
GenBank accession number	KY908400	KY908401

### Structure and gene content of the *Circaeaster* and *Kingdonia* plastomes

**Fig. 1:**
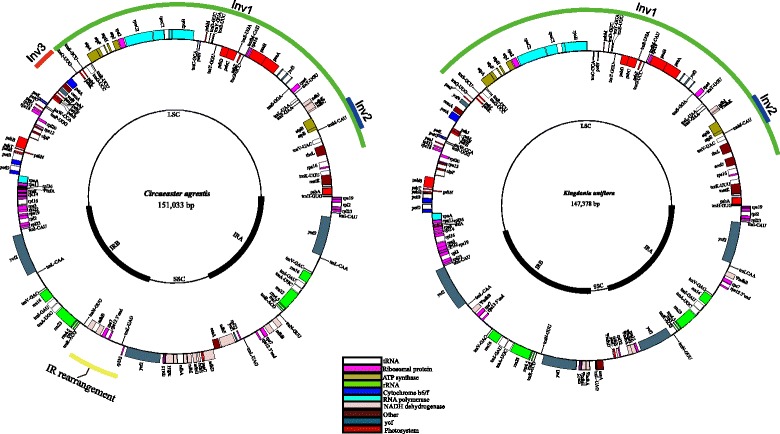
Plastome maps of *Circaeaster agrestis* and *Kingdonia uniflora.* IR, inverted repeat; LSC, large single-copy region; SSC, small single-copy region; Inv1, large inversion 1; Inv2, large inversion 2; Inv3, large inversion 3. The IR rearrangement is also indicated

**Fig. 2:**
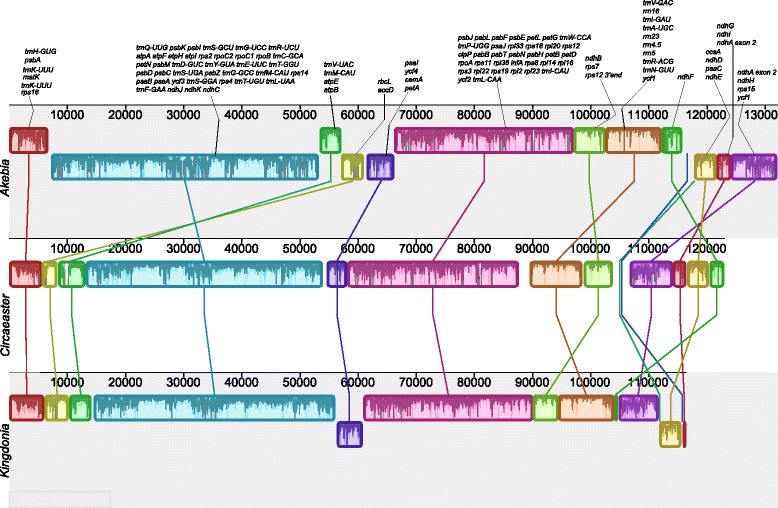
ProgressiveMauve alignment among *Akebia*, *Circaeaster* and *Kingdonia* showing the structural rearrangements in *Circaeaster* and *Kingdonia*. Colored blocks represent locally collinear blocks (LCBs) and are connected by lines to similarly colored LCBs, indicating homology

The plastome size of *Circaeaster agrestis* is 151,033 bp and that of *Kingdonia uniflora* is 147,378 bp (Fig. 1). Both plastomes possess the typical quadripartite structure of angiosperms, although both also contain several remarkable structural rearrangements. Most notably, a large ~49 kb inversion in the LSC region, including all genes from *trnQ-UUG* to *rbcL/accD* (*accD* is absent from *Circaeaster*) is present in both plastomes (Figs. 1, 2). In addition, both plastomes also share a much smaller inversion (~3.5 kb) involving all four genes from *atpB* to *trnV-UAC* (Figs. 1, 2). *Circaeaster* also possesses a number of other unique structural changes, including a ~ 3.5 kb inversion involving all four genes from *psaI* to *petA* (Figs. 1, 2) and a highly unusual IR structure. Specifically, the following changes have occurred within the IR of *Circaeaster*: (1) *ndhB*, *rps*7, and the 3′ end of *rps12* have shifted to a position between *trnN-GUU* and *ycf1* (compared to their typical positions between *trnL-CAA* and *trnV-GAC* in nearly all other angiosperms); (2) *rpl32* and *trnL-UAG* are within the IR (rather than in the SSC region as in nearly all other angiosperms), and (3) *ycf1* is almost entirely outside the IR (rather than having ~1000 bp of *ycf1* within the IR, as is more typical of angiosperms). Within *Kingdonia*, the IR/SSC boundary has shifted to include all of *ycf1, rps15, ΨndhH,* and *ΨndhA*. In both plastomes, there are unusual arrangements of *rpl32* and *trnL-UAG*, which in almost all other angiosperms are found adjacent to each other on the same strand within the SSC. The endpoints of these inversions were confirmed in both plastomes via PCR and Sanger sequencing using custom-designed primers (Additional files 1 and 2).

Overall, *Circaeaster* and *Kingdonia* were found to possess the typical gene and intron complement of angiosperms, with a few notable exceptions (Table 1). Both plastomes contain 30 tRNA genes and four rRNA genes, as is typical in angiosperms. The plastome of *Circaeaster agrestis* has 77 protein-coding genes and one pseudogene (*ΨinfA*, which is truncated to a length of 36 bp); *accD* is absent. The plastome of *Kingdonia uniflora* only has 70 protein-coding genes due to the loss or pseudogenization of nearly all *ndh* genes: four genes (*ndhC*, *ndhF*, *ndhI* and *ndhG*) were absent and five (*ΨndhA*, *ΨndhB*, *ΨndhD*, *ΨndhH* and *ΨndhK*) were identified as pseudogenes. More specifically, the second exon is absent from *ΨndhA* and *ΨndhB*, *ΨndhD* is severely truncated to 18 bp (vs. 1503 bp in *Circaeaster*), *ΨndhH* is truncated to 618 bp (vs. 1182 bp in *Circaeaster*) in length, and *ΨndhK* is only 237 bp (vs. 669 bp in *Circaeaster*) in length. The Ks values of these pseudogenes were calculated between *Circaeaster* and *Kingdonia* (Additional file 3). A total of 32 and 14 repeats ≥30 bp in length were found in the plastome of *Circaeaster* and *Kingdonia*, respectively (Additional files 4 and 5). For comparison, the number of repeats ≥30 bp in seven other Ranunculales species are as follows: (1) 17 in *Akebia trifoliata* (Thunb.) Koidz.; (2) 24 in *Epimedium sagittatum* (Sieb. & Zucc.) Maxim.; (3) 17 in *Euptelea pleiosperma* Hook.f. & Thomson; (4) 29 in *Mahonia bealei* (Fortune) Pynaert; (5) nine in *Nandina domestica* Thunb.; (6) nine in *Papaver somniferum* L.; and (7) eight in *Stephania japonica* (Thunb.) Miers (Additional file 6)*.*

### Phylogenetic analyses

**Fig. 3:**
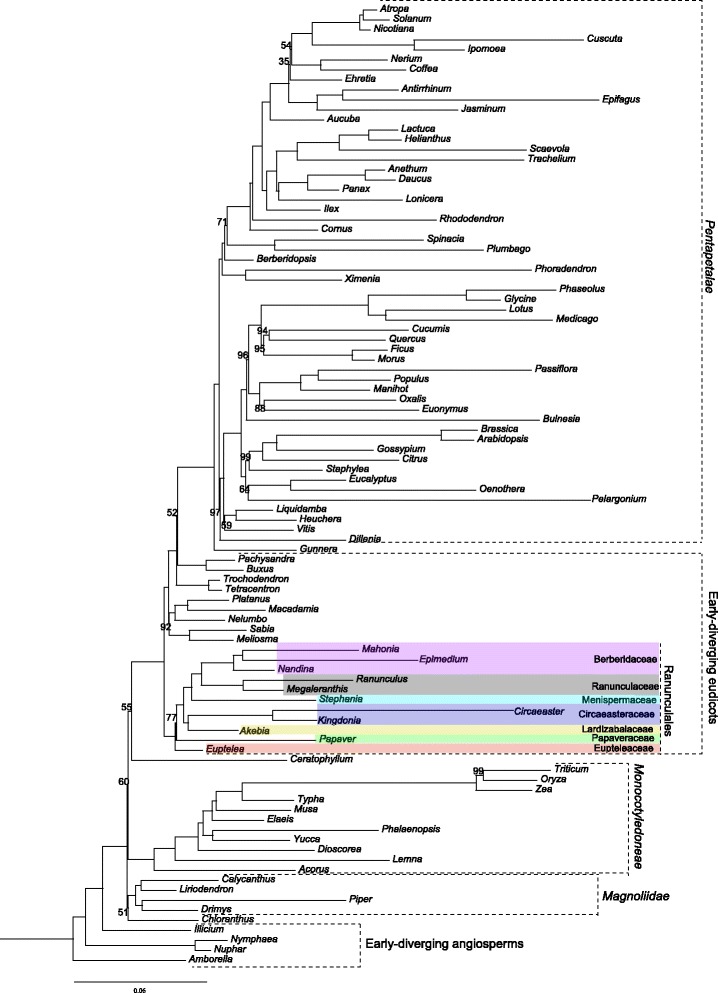
Phylogram of the best tree determined by RAxML for the 79-gene, 99-taxon data set using the 35-partition scheme recovered as optimal by PartitionFinder. Numbers associated with branches are ML bootstrap support values. Branches with no bootstrap values listed have 100% bootstrap support

The final 79-gene, 99-taxon alignment used for ML analyses was 62,238 bp in length after character exclusion. The best partitioning scheme identified under the Bayesian information criteria (BIC) using relaxed clustering analysis in PartitionFinder (ln *L =* −1,173,388.00241; BIC 2353123.26941) contained 35 partitions. The tree with the highest ML score (ln *L* = −1,178,285.119460) produced by the 35-partition ML analysis (Fig. 3) shared an identical topology with the best tree from unpartitioned analysis (ln *L* = −1,200,753.541175) (Additional file 7), except for the relationships among Trochodendrales, Buxales and Gunneridae. The 35-partition analysis supported the sister relationship between Buxales and Gunneridae, but the support value was low (52%); while the unpartitioned analysis strongly supported the sister relationship between Trochodendrales and Gunneridae. Within Ranunculales, Eupteleaceae was found to be the earliest-diverging lineage, and Papaveraceae was sister to the clade comprised of Berberidaceae, Ranunculaceae, Menispermaceae, Lardizabalaceae and Circaeasteraceae. Lardizabalaceae and Circaeasteraceae formed a strongly supported clade that was sister to the clade of Berberidaceae, Ranunculaceae and Menispermaceae.

## Discussion

### Plastome structure and gene content

The unusual structural rearrangements and gene losses (especially the loss of the *ndh* genes) detected in the two Circaeasteraceae plastomes represent the first reported among early-diverging eudicot plastomes, and hence shed important insight into the evolution of early eudicot plastomes. The fact that two of the observed inversions (the ~49 kb and ~3.5 kb inversions) are shared by *Circaeaster* and *Kingdonia* suggests that they predate the evolutionary split between these two genera. Although uncommon, relatively large inversions have been detected in a number of other angiosperm lineages and often serve as useful phylogenetic markers [50, 52–54]. Some of the best examples of relatively large inversions that are synapomorphies for clades of flowering plants include the 22.8 kb inversion shared by all Asteraceae except Barnadesioideae [53, 55], the 78 kb inversion shared by all Fabaceae subtribe Phaseolinae [56], and the 36 kb inversion present in all core genistoid legumes [50, 57]. Highly rearranged plastome structures also characterize a number of other angiosperm lineages, such as Campanulaceae, Geraniaceae, and the IR-lacking clade of Fabaceae, and these are associated with greatly elevated rates of molecular evolution and large numbers of short repeats [58]. In some cases the endpoints of large plastome inversions have been found to be associated with short inverted repeats (sIRs), although we did not detect sIRs in association with the inversion endpoints in *Circaeaster* or *Kingdonia*.

The IR regions of *Circaeaster* and *Kingdonia* are also structurally unique among angiosperms, with several rearrangements. The most unusual of these involves the transposition of the *ndhB, rps7* and 3′ end of the *rps12* gene to a point near the junction of the IR_B_ and the SSC region (Fig. 1). These three genes form a transcriptional operon [59] and this operon is not disrupted in *Circaeaster*, nor does its transposition interrupt adjacent operons. The IR/SSC endpoints themselves are also rearranged in *Circaeaster*, with *rpl32* and *trnL-UAG* within the IR and non-adjacent to *ndhF*, unlike almost all other angiosperms [54]. The IR boundaries of *Kingdonia* are also unusual for their expansion to include several genes that are normally in the SSC (e.g. *ycf1, rps15*), resulting in a much smaller and rearranged SSC (which is also influenced by the loss of *ndh* genes; see below). The exact sequence of rearrangements that could account for the unusual IR arrangements of Circaeasteraceae is clearly complicated and hence difficult to reconstruct. IR expansion and contraction is well-known in a number of other angiosperm lineages (e.g. [45]), including within early-diverging eudicots, which have been found to possess a number of expansions and contractions [23]. Given the expansions and rearrangements observed in *Circaeaster* and *Kingdonia*, neither fit into any of the six IR types for early-diverging eudicots delineated in Sun et al. [23], and thus we designate two new early-diverging eudicot IR types for *Circaeaster* (type G) and *Kingdonia* (type H) (Fig. 1).

Usually, gene content is highly conserved among photosynthetic angiosperm plastomes [50, 60], but in Circaeasteraceae, a number of genes have been lost or pseudogenized, each of which has been found to be lost repeatedly across angiosperms. For example, the loss of *accD in Circaeaster* is mirrored in a number of other lineages where *accD* is pseudogenized or absent, e.g., grasses [61], Lobeliaceae [62], Campanulaceae [52, 63, 64], *Acorus* [65], Oleaceae [66], *Pelargonium* [67], and *Lolium perenne* [68]. Likewise, *infA* is also known to be a pseudogene in numerous other angiosperms, including two Ranunculales (*Ranunculus macranthus* and *Epimedium sagittatum*) [23], tobacco [69], and numerous rosids [70]. Whether *accD* or *infA* have been transferred to the nucleus in *Circaeaster* is unknown.

The loss or pseudogenization of nearly all *ndh* genes from *Kingdonia* has also been observed in a number of other land plants. The *ndh* genes encode subunits of the plastid NDH (NADH dehydrogenase-like) complex, which permits cyclic electron flow associated with Photosystem I and hence facilitates chlororespiration [71, 72]. Although the NDH complex is widely retained across land plants, it has been found to be dispensable under optimal growth conditions and the plastid *ndh* genes have been lost in a number of autotrophic and heterotrophic lineages [72, 73]. For example, the plastid *ndh* loci have been lost or pseudogenized en masse from parasitic plants such as Orobanchaceae and *Cuscuta* (Convolvulaceae) [74–76], from mycoheterotrophs including several orchids [77] and *Petrosavia* (Petrosaviaceae) [78], and from autotrophs including Gnetales, conifers, *Najas* (Hydrocharitaceae), *Carnegiea* (Cactaceae), and *Erodium* (Geraniaceae) [79–84]. It is not clear whether the *ndh* genes in *Kingdonia* have been transferred to the nucleus or whether their loss represents the complete loss of the NDH complex, but in any case *Kingdonia* is the only known early-diverging eudicot that has experienced *ndh* pseudogenization and loss.

Moreover, the loss of the *ndh* genes in *Kingdonia* accounts for the smaller overall size of the *Kingdonia* plastome and may have also played an indirect role in the expansion of the IR of *Kingdonia*. The complete loss of *ndhF*, which normally occupies a position immediately adjacent to the IR_B_/SSC boundary, may have led to instability of the IR/SSC boundaries, leading to rearrangements of the SSC and IR. This hypothesis is supported by other recent studies in orchids [77] and *Najas flexilis* [82] where *ndhF* loss is associated with shifts in the IR/SSC boundary.

### Phylogeny of Ranunculales

The circumscription of Ranunculales was long controversial (e.g. [3, 4, 9, 10, 85, 86]), but molecular phylogenetics has clarified the delimitation of Ranunculales to Berberidaceae, Circaeasteraceae, Eupteleaceae, Lardizabalaceae, Menispermaceae, Papaveraceae, and Ranunculaceae [1, 5, 6, 26, 29, 31, 32, 36, 38]. While the expansion of Circaeasteraceae to include *Kingdonia* is accepted by a majority of taxonomists [1], the rank and position of *Kingdonia* have long been in dispute [38]. The complete plastome sequence data strongly support the sister relationship between *Kingdonia* and *Circaeaster*, in concordance with previous molecular results [25, 26, 31, 32, 36, 38, 87]. The two inversions and the rare, open dichotomous leaf venation shared by these taxa are good synapomorphies that additionally support the placement of *Circaeaster* and *Kingdonia* in one family.

## Conclusions

The plastomes of *Circaeaster agrestis* and *Kingdonia uniflora* provide the first reference genome sequences for Circaeasteraceae, which will enrich the sequence resources of plastomes in early-diverging eudicots. The unusual rearrangements including large inversions and unusual IR structure detected in the Circaeasteraceae plastomes will help us better characterize plastome structural evolution in early-diverging eudicots. Phylogenetic analyses of the 79-gene, 99-taxon data set confirmed the position of Circaeasteraceae in Ranunculales, with maximum support as sister to Lardizabalaceae. The two Circaeasteraceae plastomes will also be of benefit for further phylogenomic analyses within early-diverging eudicots.

## Methods

### Taxon sampling, chloroplast DNA isolation, high-throughput sequencing

Fresh leaves of *Circaeaster agrestis* were collected from Shennongjia, Hubei Province, China, in 2015, and from *Kingdonia uniflora* in Meixian, Shanxi Province, China, in 2016. Voucher specimens (*Circaeaster agrestis*: Y.X. Sun 1510; *Kingdonia uniflora*: Y.X. Sun 1606) were deposited at the Herbarium of Wuhan Botanical Garden, Chinese Academy of Sciences (HIB). For both species, high-quality chloroplast DNA was obtained following the plastid DNA extraction method of Shi et al. [88]. The sequencing libraries were constructed and quantified following the methods introduced by Sun et al. [23]. For both plastomes, a 500-bp DNA TruSeq Illumina (Illumina Inc., San Diego, CA, USA) sequencing library was constructed using 2.5–5.0 ng sonicated chloroplast DNA as input. Libraries were quantified using an Agilent 2100 Bioanalyzer (Agilent Technologies, Santa Clara, CA, USA) and by real-time quantitative PCR. Libraries were then multiplexed, and 2 × 125 bp sequencing was performed on an Illumina HiSeq 2000 platform at the Beijing Genomics Institute.

### Plastome assembly, annotation, and structural analyses

Following Sun et al. [23], duplicate reads, adapter-contaminated reads, and reads with more than five Ns were filtered out. Remaining, high-quality reads were assembled into contigs with a minimum length of 1000 bp using CLC Genomics Workbench with default parameters, except for a word size value of 60.

Plastomes were annotated using DOGMA [89] and through comparison with the sequences of published early-diverging eudicot plastomes. Physical maps were drawn using GenomeVx [90], followed by subsequent manual editing with Adobe Illustrator CS5. Boundaries for tRNAs were identified with tRNAscan-SE 1.21 [91] and confirmed by comparison with available early-diverging eudicot plastome sequences. The finished genomes were deposited in GenBank (Table 1).

To investigate plastome structural evolution, whole plastome alignment between Circaeasteraceae and representatives of other early-diverging eudicot families was performed with ProgressiveMauve v 2.4.0 [92], including only one copy of the IR (IR_B_), and locally collinear blocks (LCBs) were identified. Because the 18 reported early-diverging eudicot plastomes in Sun et al. [23] share the same gene order, and because Circaeasteraceae was resolved as sister to *Akebia* in present research, the *Akebia* plastome was used as the reference sequence for ProgressiveMauve comparisons*.* mVISTA [93] was employed to generate sequence identity plots. The number and location of repeat elements in the plastomes of *Circaeaster* and *Kingdonia* as well as seven other Ranunculales species (*Akebia trifoliata, Epimedium sagittatum*, *Euptelea pleiosperma*, *Berberis bealei*, *Nandina domestica*, *Papaver somniferum* and *Stephania japonica*) were determined by REPuter [94], with a minimum size of 30 bp and a Hamming distance of 1. Before performing the analysis, one copy of the IR was removed.

### Phylogenetic analyses

All protein-coding regions were extracted from the plastomes of *Circaeaster* and *Kingdonia*. These sequences were then added manually to the 97-taxon alignment of Sun et al. [23], resulting in a data set with complete coverage of early-diverging eudicot families sensu APG IV [1]. GenBank information for all plastomes used for present phylogenetic analyses can be found in Additional file 8. Regions of ambiguous alignment and sites with more than 80% missing data were excluded from the alignment.

Maximum likelihood (ML) analyses were conducted using RAxML version 7.4.2 [95], under the general time-reversible (GTR) substitution model and the Γ model of rate heterogeneity. We conducted both unpartitioned and partitioned analyses. PartitionFinder version 1.1.1 [96] was used to select the best-fit partitioning scheme, considering all 237 possible gene-by-codon position partitions (79 genes × 3 codon positions). For both ML analyses, a single set of branch lengths for all partitions was used. Ten independent ML searches were conducted and bootstrap support was estimated with 1000 bootstrap replicates.

## Additional files

Additional file 1: Sanger chromatograms of primer products C1-C11 and K1-K8. (ZIP 2226 kb)

Additional file 2: Primers designed for verifying endpoints of plastome structural rearrangements. (DOC 54 kb)

Additional file 3: Ks values calculation of five pseudogenes in *Kingdonia. *(DOC 29 kb)

Additional file 4: Repeats ≥30 bp in the plastome of *Circaeaster.* F, forward; P, palindromic. (DOC 51 kb)

Additional file 5: Repeats ≥30 bp in the plastome of *Kingdonia*. F, forward; P, palindromic. (DOC 42 kb)

Additional file 6: Repeats ≥30 bp in the plastomes of seven other Ranunculales species. (DOC 30 kb)

Additional file 7: Phylogram of the best tree determined by RAxML for the 79-gene, 99-taxon data set with no data partitions. Numbers associated with branches are ML bootstrap support values. Branches with no bootstrap values listed have 100% bootstrap support. (PDF 204 kb)

Additional file 8: List of taxa included in phylogenetic analyses. (DOC 67 kb)
