# Supplementary material for: Complete plastome sequencing of both living species of Circaeasteraceae (Ranunculales) reveals unusual rearrangements and the loss of the ndh gene family
Source: BMC Genomics. 2017 Aug 9;18:592. doi: 10.1186/s12864-017-3956-3 (PMC5551029; doi:10.1186/s12864-017-3956-3)
Supplement: Supplementary file 2 — Primers designed for verifying endpoints of plastome structural rearrangements. (DOC 54 kb) [file 12864_2017_3956_MOESM2_ESM.doc]

Additional file 2 Primers designed for verifying endpoints of plastome structural rearrangements.

| Structural rearrangement | |  | Primer name | Primer sequence (5'🡪3') | Location | PCR product |
| --- | --- | --- | --- | --- | --- | --- |
| Inv1 |  | *Circaeaster* | CF1 | GTTGCTTTCTACCACATCGTTT | *rps16* exon 1 | C1 |
|  |  |  | CR1 | TCGAATTTGAAGCAATGGATACTT | *rbcL* |  |
|  | Inv3 |  | CF2 | CGTAGCCAAGTGGTAAGGCA | *trnQ-UUG* | C2 |
|  |  |  | CR2 | AGTTTGAGAAGGTTCAATTATCCGA | *petA* |  |
|  |  |  | CF3 | CAAGTGCCGGAAATACTAGGC | *psaI* | C3 |
|  |  |  | CR3 | GCTGATAGGTACTGTAGCTGGT | *psbJ* |  |
| Inv2 | |  | CF4 | CCAGGACCGGAAGGAGTAGG | *atpB* | C4 |
|  |  |  | CR4 | GGTCCTTCGTTAATCGGGGC | *ndhC* |  |
|  |  |  | CF5 | TGCTTCTTCAGGTGGAACCC | *rbcL* | C5 |
|  |  |  | CR5 | CGGTTCGAATCCGTATAGCCC | *trnV-UAC* exton 2 |  |
| IR Rearrangement | |  | CF6 | CGCTCTACCACCGAGCTACT | *trnN-GUU* | C6 |
|  |  |  | CR6 | CCAAGAAATAACCCCGCACG | *ndhB* exton 2 |  |
|  |  |  | CF7 | CTCGGACTCGAACCGAGATG | *trnL-UAG* | C7 |
|  |  |  | CR7 | TTTCCGGTAGCCAGCGATTT | *rpl32* |  |
| Inv1 | | *Kingdonia* | KF1 | ATCATGTCCTTCAAGCCGCA | *rps16* intron | K1 |
|  |  |  | KR1 | TGAGTGAGTTATTTAAGCTTCA | *accD* |  |
|  |  |  | KF2 | CCTTCCGTCCCAGAACAGTC | *trnQ-UUG* | K2 |
|  |  |  | KR2 | CCATTGCAAGTGCCGGAAATA | *psaI* |  |
| Inv2 | |  | KF3 | CTTCAAGTGCGGAAACCCCA | *atpB* | K3 |
|  |  |  | KR3 | CGATTCTCCTGTTGAAACCCAA | *ndhK* |  |
|  |  |  | KF4 | CCCGCTTTGAATCCAACACTT | *rbcL* | K4 |
|  |  |  | KR4 | CGGTTCGAATCCGTATAGCC | *trnV-UAC* exton 2 |  |
