# Supplementary material for: Complete plastome sequencing of both living species of Circaeasteraceae (Ranunculales) reveals unusual rearrangements and the loss of the ndh gene family
Source: BMC Genomics. 2017 Aug 9;18:592. doi: 10.1186/s12864-017-3956-3 (PMC5551029; doi:10.1186/s12864-017-3956-3)
Supplement: Supplementary file 3 — Ks values calculation of five pseudogenes in Kingdonia. (DOC 29 kb) [file 12864_2017_3956_MOESM3_ESM.doc]

*Additional file 3 Ks values calculation of five pseudogenes in Kingdonia*

| Taxa | pseudogene | Ks |
| --- | --- | --- |
| *Circaeaster/Kingdonia* | *ΨndhA* | 0.2729 |
| *Circaeaster/Kingdonia* | *ΨndhB* | 0.0484 |
| *Circaeaster/Kingdonia* | *ΨndhD* | 0.0000 |
| *Circaeaster/Kingdonia* | *ΨndhH* | 0.2838 |
| *Circaeaster/Kingdonia* | *ΨndhK* | 0.2079 |
