# Supplementary material for: Complete plastome sequencing of both living species of Circaeasteraceae (Ranunculales) reveals unusual rearrangements and the loss of the ndh gene family
Source: BMC Genomics. 2017 Aug 9;18:592. doi: 10.1186/s12864-017-3956-3 (PMC5551029; doi:10.1186/s12864-017-3956-3)
Supplement: Supplementary file 4 — Repeats ≥30 bp in the plastome of Circaeaster. F, forward; P, palindromic. (DOC 51 kb) [file 12864_2017_3956_MOESM4_ESM.doc]

Additional file 4 Repeats ≥ 30 bp in the plastome of *Circaeaster.* F, forward; P, palindromic.

| Start of the first repeat | Start of the second repeat | Number of mismatches between repeats | Repetition type | Motif size |
| --- | --- | --- | --- | --- |
| 89243 | 105605 | 1 | F | 85 |
| 88302 | 105971 | 1 | F | 84 |
| 46976 | 46976 | 0 | P | 64 |
| 16486 | 104833 | 1 | P | 59 |
| 16547 | 104775 | 1 | p | 58 |
| 89590 | 104642 | 0 | F | 54 |
| 54080 | 88719 | 1 | P | 56 |
| 80097 | 87781 | 0 | P | 51 |
| 98259 | 98367 | 1 | F | 48 |
| 19716 | 103017 | 0 | P | 39 |
| 16585 | 104755 | 1 | P | 40 |
| 81226 | 81310 | 1 | F | 40 |
| 80055 | 87836 | 1 | P | 38 |
| 98847 | 104010 | 1 | P | 37 |
| 87330 | 89499 | 1 | F | 35 |
| 98293 | 98401 | 1 | F | 35 |
| 80915 | 88257 | 1 | F | 34 |
| 89208 | 105570 | 1 | F | 34 |
| 88481 | 106150 | 0 | F | 30 |
| 16519 | 104827 | 1 | P | 32 |
| 53873 | 89262 | 1 | P | 32 |
| 53873 | 105624 | 1 | P | 32 |
| 87318 | 89616 | 1 | F | 32 |
| 88925 | 106603 | 1 | F | 32 |
| 122789 | 122827 | 1 | P | 32 |
| 80806 | 105866 | 1 | F | 31 |
| 89435 | 104011 | 1 | F | 31 |
| 18129 | 52743 | 1 | P | 30 |
| 83029 | 83050 | 1 | F | 30 |
| 88755 | 106442 | 1 | F | 30 |
| 98829 | 104070 | 1 | P | 30 |
| 104531 | 104551 | 1 | F | 30 |
