# Supplementary material for: Complete plastome sequencing of both living species of Circaeasteraceae (Ranunculales) reveals unusual rearrangements and the loss of the ndh gene family
Source: BMC Genomics. 2017 Aug 9;18:592. doi: 10.1186/s12864-017-3956-3 (PMC5551029; doi:10.1186/s12864-017-3956-3)
Supplement: Supplementary file 5 — Repeats ≥30 bp in the plastome of Kingdonia. F, forward; P, palindromic. (DOC 42 kb) [file 12864_2017_3956_MOESM5_ESM.doc]

Additional file 5 Repeats ≥ 30 bp in the plastome of *Kingdonia*. F, forward; P, palindromic.

| Start of the first repeat | Start of the second repeat | Number of mismatches between repeats | Repetition type | Motif size |
| --- | --- | --- | --- | --- |
| 94041 | 103674 | 1 | F | 95 |
| 108178 | 108208 | 0 | F | 60 |
| 5199 | 56240 | 1 | P | 43 |
| 20707 | 93324 | 0 | P | 39 |
| 27978 | 28013 | 0 | F | 37 |
| 94100 | 103733 | 0 | F | 36 |
| 6269 | 6286 | 0 | F | 33 |
| 32480 | 32529 | 0 | P | 33 |
| 61211 | 61258 | 0 | P | 33 |
| 5183 | 56263 | 1 | P | 36 |
| 108178 | 108178 | 0 | F | 30 |
| 89959 | 96956 | 1 | P | 33 |
| 85216 | 85237 | 1 | F | 31 |
| 18995 | 54823 | 1 | P | 30 |
