# Supplementary material for: Complete plastome sequencing of both living species of Circaeasteraceae (Ranunculales) reveals unusual rearrangements and the loss of the ndh gene family
Source: BMC Genomics. 2017 Aug 9;18:592. doi: 10.1186/s12864-017-3956-3 (PMC5551029; doi:10.1186/s12864-017-3956-3)
Supplement: Supplementary file 6 — Repeats ≥30 bp in the plastomes of seven other Ranunculales species. (DOC 30 kb) [file 12864_2017_3956_MOESM6_ESM.doc]

Additional file 6 Repeats ≥ 30 bp in the plastomes of seven other Ranunculales species.

| Taxa | Number repeats |
| --- | --- |
| *Akebia trifoliata* | 17 |
| *Epimedium sagittatum* | 24 |
| *Euptelea pleiosperma* | 17 |
| *Berberis bealei* | 29 |
| *Nandina domestica*, | 9 |
| *Papaver somniferum* | 9 |
| Stephania *japonica* | 8 |
