# Supplementary figures and images for: Complete plastome sequencing of both living species of Circaeasteraceae (Ranunculales) reveals unusual rearrangements and the loss of the ndh gene family
Source: BMC Genomics. 2017 Aug 9;18:592. doi: 10.1186/s12864-017-3956-3 (PMC5551029; doi:10.1186/s12864-017-3956-3)

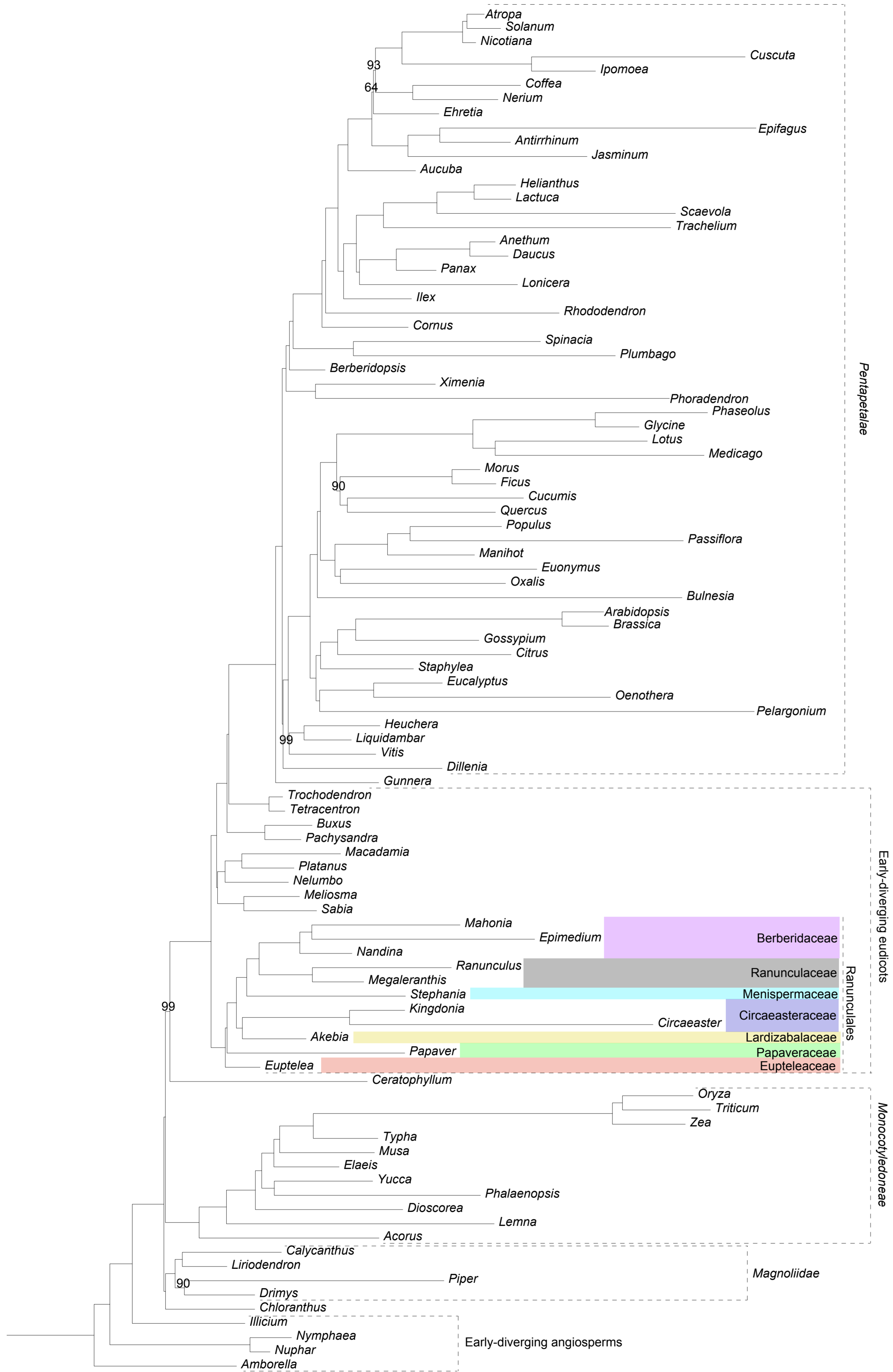

Supplement: Supplementary file 7 — Phylogram of the best tree determined by RAxML for the 79-gene, 99-taxon data set with no data partitions. Numbers associated with branches are ML bootstrap support values. Branches with no bootstrap values listed have 100% bootstrap support. (PDF 204 kb) [file 12864_2017_3956_MOESM7_ESM.pdf]
