# Supplementary material for: Complete plastome sequencing of both living species of Circaeasteraceae (Ranunculales) reveals unusual rearrangements and the loss of the ndh gene family
Source: BMC Genomics. 2017 Aug 9;18:592. doi: 10.1186/s12864-017-3956-3 (PMC5551029; doi:10.1186/s12864-017-3956-3)
Supplement: Supplementary file 8 — List of taxa included in phylogenetic analyses. (DOC 67 kb) [file 12864_2017_3956_MOESM8_ESM.doc]

Additional file 8 List of taxa included in phylogenetic analyses

| Taxon | GenBank Accession Number(s) |
| --- | --- |
| *Cycas micronesica* K.D.Hill | EU016802-EU016882 |
| *Ginkgo biloba* L. | DQ069337-DQ069702, EU016963-EU016982 |
| *Pinus thunbergii* Parl. | NC_001631 |
| *Amborella trichopoda* Baill. | NC_005086 |
| *Illicium oligandrum* Merr. & Chun | NC_009600 |
| *Nuphar advena* Ait. | NC_008788 |
| *Nymphaea alba* L. | NC_006050 |
| *Ceratophyllum demersum* L. | NC_009962 |
| *Chloranthus spicatus* Makino | NC_009598 |
| *Akebia trifoliata* (Thunb.) Koidz. | KU204898 |
| *Anethum graveolens* L. | EU016721-EU016801 |
| *Antirrhinum majus* L. | GQ996966-GQ997048 |
| *Arabidopsis thaliana* (L.) Heynh. | NC_000932 |
| *Atropa belladonna* L. | NC_004561 |
| *Aucuba japonica* Thunb. | GQ997049-GQ997131 |
| *Berberidopsis corallina* Hook.f. | EU002158, EU002171,  EU002201,  EU002274,  EU002295, EU002385,  EU002466, GQ997932-GQ998004 |
| *Brassica rapa* L. | DQ231548 |
| *Bulnesia arborea* (Jacq.) Engl. | EU002159, EU002172, EU002205, EU002275, EU002299, EU002388, EU002478, GQ998005-GQ998073 |
| *Buxus microphylla* Siebold & Zucc. | NC_009599 |
| *Circaeaster* *agrestis* Maxim. | KY908400 |
| *Citrus sinensis* Osbeck | NC_008334 |
| *Coffea arabica* L. | NC_008535 |
| *Cornus florida* L. | EU002157, EU002175, EU002215, EU002276, EU002311, EU002377, EU002491, GQ998074-GQ998146 |
| *Cucumis sativus* L. | NC_007144 |
| *Cuscuta exaltata* Engelm. | NC_009963 |
| *Daucus carota* L. | NC_008325 |
| *Dillenia indica* L. | GQ997132-GQ997214 |
| *Ehretia acuminata* R.Br. | GQ997215-GQ997297 |
| *Epifagus virginiana* (L.) W.P.C.Barton | NC_001568 |
| *Epimedium sagittatum* (Sieb. & Zucc.) Maxim. | KU204899 |
| *Eucalyptus globulus* Labill. | NC_008115 |
| *Euonymus americanus* L. | EU002160, EU002170, EU002193, EU002277, EU002321, EU002409, EU002500, GQ998147-GQ998219 |
| *Euptelea pleiosperma* Hook.f. & Thomson | KU204900 |
| *Ficus* sp. | EU002161, EU002177, EU002224, EU002278, EU002322, EU002410, EU002501, GQ998220-GQ998291 |
| *Glycine max* Merr. | NC_007942 |
| *Gossypium hirsutum* L. | NC_007944 |
| *Gunnera manicata* Linden ex André | EU002162, EU002179, EU002226, EU002279, EU002325, EU002413, EU002504, GQ998292-GQ998364 |
| *Helianthus annuus* L. | NC_007977 |
| *Heuchera sanguinea* Engelm. | EU002163, EU002180, EU002228, EU002280, EU002327, EU002415, EU002506, GQ998365-GQ998437 |
| *Ilex cornuta* Lindl. & Paxton | GQ997298-GQ997380 |
| *Ipomoea purpurea* (L.) Roth | NC_009808 |
| *Jasminum nudiflorum* Lindl. | NC_008407 |
| *Kingdonia* *uniflora* Balf.f. & W.W. Smith | KY908401 |
| *Lactuca sativa* L. | NC_007578 |
| *Liquidambar styraciflua* L. | EU002164, EU002182, EU002239, EU002281, EU002341, EU002429, EU002519, GQ998438-GQ998510 |
| *Lonicera japonica* Thunb. | GQ997381-GQ997463 |
| *Lotus corniculatus* L. | NC_002694 |
| *Macadamia integrifolia* Maiden & Betche | NC_025288 |
| *Mahonia bealei* (Fortune) Pynaert | NC_022457 |
| *Manihot esculenta* Crantz | NC_010433 |
| *Medicago truncatula* Gaertn. | NC_003119 |
| *Megaleranthis saniculifolia* Ohwi | NC_012615 |
| *Meliosma* aff. *cuneifolia* Franch. | GQ997464-GQ997546 |
| *Morus indica* L. | NC_008359 |
| *Nandina domestica* Thunb. | NC_008336 |
| *Nelumbo nucifera* Gaertn. | GQ997547-GQ997629 |
| *Nerium oleander* L. | GQ997630-GQ997712 |
| *Nicotiana tabacum* L. | NC_001879 |
| *Oenothera elata* Kunth | NC_002693 |
| *Oxalis latifolia* Kunth | EU002165, EU002186, EU002248, EU002282, EU002350, EU002438, EU002528, GQ998511-GQ998580 |
| *Pachysandra terminalis* Siebold & Zucc. | KU204904 |
| *Panax ginseng* C.A.Mey. | NC_006290 |
| *Papaver somniferum* L. | KU204905 |
| *Passiflora biflora* Lam. | EU017064-EU017138 |
| *Pelargonium* × *hortorum* L.H. Bailey | NC_008454 |
| *Phaseolus vulgaris* L. | EU196765 |
| *Phoradendron leucarpum* (Raf.) Reveal & M.C.Johnst. | GQ997713-GQ997781 |
| *Platanus occidentalis* L. | NC_008335 |
| *Plumbago auriculata* Lam. | EU002166, EU002187, EU002252, EU002283, EU002354, EU002442, EU002532, GQ998581-GQ998652 |
| *Populus alba* L. | NC_008235 |
| *Quercus nigra* L. | EU002167, EU002188, EU002254, EU002284, EU002357, EU002445, EU002535, GQ998653-GQ998724 |
| *Ranunculus macranthus* Scheele | NC_008796 |
| *Rhododendron simsii* Planch. | GQ997782-GQ997859 |
| *Sabia yunnanensis* Franch. | KU204902 |
| *Scaevola aemula* R.Br. | EU017139-EU017217 |
| *Solanum lycopersicum* L. | DQ347959 |
| *Spinacia oleracea* L. | NC_002202 |
| *Staphylea colchica* Steven | EU002168, EU002189, EU002261, EU002285, EU002364, EU002453, EU002543, GQ998725-GQ998796 |
| *Stephania japonica* (Thunb.) Miers | KU204903 |
| *Tetracentron sinense* Oliv. | NC_021425 |
| *Trachelium caeruleum* L. | NC_010442 |
| *Trochodendron aralioides* Siebold & Zucc. | EU002169, EU002269, EU002372, EU002461, EU002551, GQ998797-GQ998871 |
| *Vitis vinifera* L. | NC_007957 |
| *Ximenia americana* L. | GQ997860-GQ997931 |
| *Calycanthus floridus* L. | NC_004993 |
| *Drimys granadensis* L.f. | NC_008456 |
| *Liriodendron tulipifera* L. | NC_008326 |
| *Piper cenocladum* C.DC. | NC_008457 |
| *Acorus americanus* Raf. | DQ069337-DQ069702, EU0167701-EU016720 |
| *Dioscorea elephantipes* Engl. | NC_009601 |
| *Elaeis oleifera* (Kunth) Cortés ex Prain | EU016883-EU016962 |
| *Lemna minor* L. | NC_010109 |
| *Musa acuminata* Colla | EU016983-EU017063 |
| *Oryza sativa* L. | NC_001320 |
| *Phalaenopsis aphrodite* Rchb.f. | NC_007499 |
| *Triticum aestivum* L. | NC_002762 |
| *Typha latifolia* L. | DQ069337-DQ069702, EU017296-EU017315 |
| *Yucca schidigera* Ortgies | DQ069337-DQ069702, EU016681-EU016700 |
